# Supplementary material for: Inflammatory factors and risk of lung adenocarcinoma: a Mendelian randomization study mediated by blood metabolites
Source: Front Endocrinol (Lausanne). 2024 Aug 27;15:1446863. doi: 10.3389/fendo.2024.1446863 (PMC11384989; doi:10.3389/fendo.2024.1446863)
Supplement: Supplementary file 1 [file DataSheet1.zip › supplementary files/S2/GCST90200535/forest.pdf]

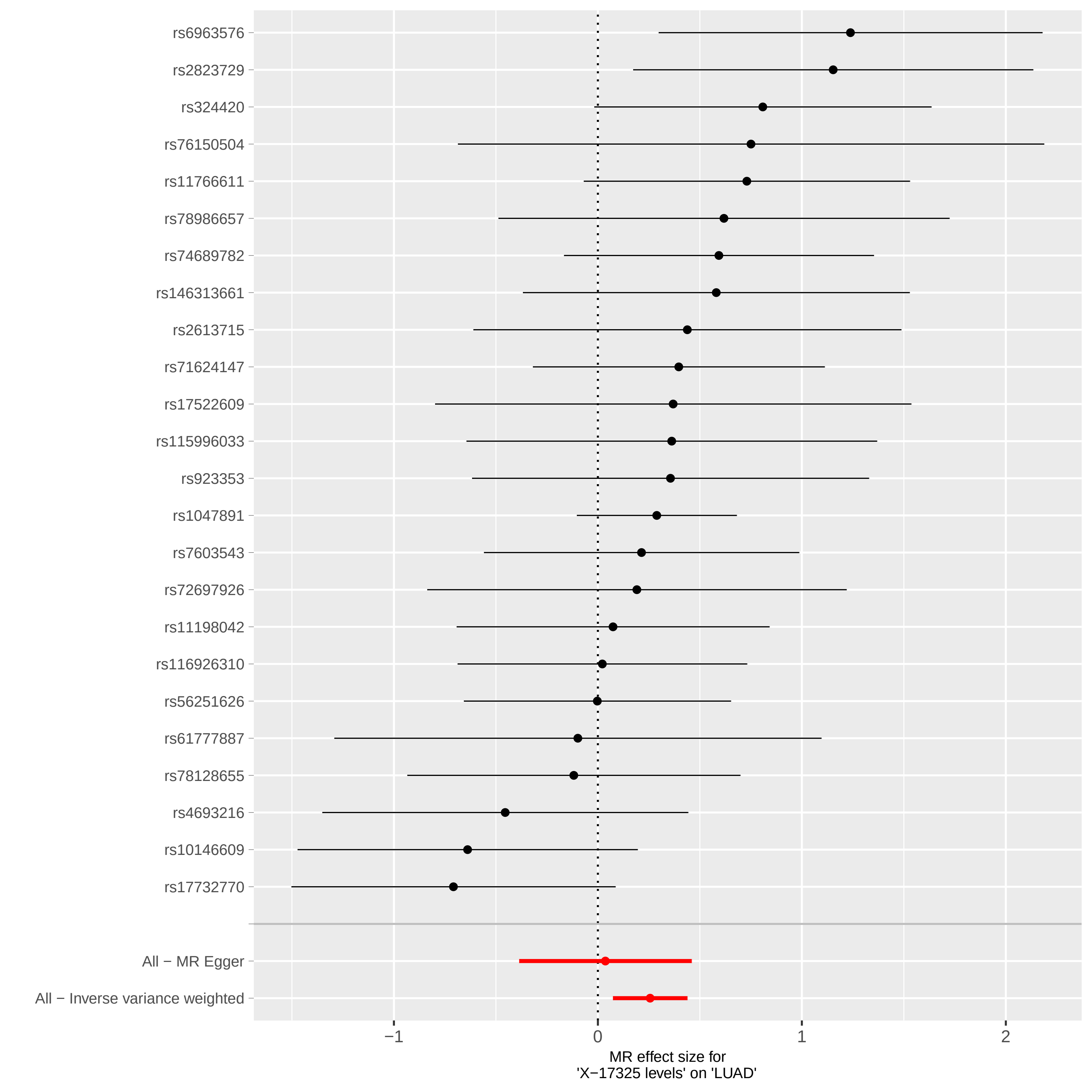

rs6963576
rs2823729
rs324420
rs76150504
rs11766611
rs78986657
rs74689782
rs146313661
rs2613715
rs71624147
rs17522609
rs115996033
rs923353
rs1047891
rs7603543
rs72697926
rs11198042
rs116926310
rs56251626
rs61777887
rs78128655
rs4693216
rs10146609
rs17732770
All − MR Egger
All − Inverse variance weighted
−1
0
1
2
MR effect size for
'X−17325 levels' on 'LUAD'
